# Supplementary material for: Beneficial Use Impairments, Degradation of Aesthetics, and Human Health: A Review
Source: Int J Environ Res Public Health. 2022 May 17;19(10):6090. doi: 10.3390/ijerph19106090 (PMC9142078; doi:10.3390/ijerph19106090)
Supplement: Supplementary file 1 [file ijerph-19-06090-s001.zip › ijerph-1697417-supplementary.pdf]

Beneficial Use Impairments, Degradation of Aesthetics, and Human Health: A Review
Supplemental Materials

Supplementary Document S1: List of evaluation domains and criteria questions
Domain 1: Population Selection
Is the population well suited for studying exposure to aesthetic degradation?
Is the population section, recruitment, inclusion/exclusion, etc., given in sufficient detail?
Are there sufficient numbers of included population to observe associations?

Domain 2: Exposure
Were there quantitative approaches to describe the aesthetic condition?
Was aesthetic condition defined, and captured in a way consistent with that definition?
Are sub-types of habitats and associated areas described?
If the study examined green/blue space, was this examined beyond the presence or absence of that space?
Is the exposure environment/controls appropriate to test the experience? Is there an exposure control/negative exposure?

Domain 3: Outcome
Is there measurement of a health outcome as opposed to an assessment of risk or hazard?
Is there clear mode of action laid out for exposure to impact health?
Is the outcome measured appropriately? Is the outcome measure specific and unlikely to be misclassified? Is there a temporal component to the outcome measure in regard to the exposure?

Domain 4: Analysis
Are appropriate confounders considered and accounted for?
Are the methods used in modeling appropriate?
Does the study design support whether the effect is based on relative state of physical space or absolute quality of space?

Supplement Document S2: Extraction table
Table S1: search terms used

Database	Search Strategy
**PubMed**	("water"[tw] OR "waterway"[tw] OR "marine"[tw] AND ("clarity"[tw] OR "color"[tw] OR "presence of debris"[tw] OR "litter"[tw] OR "trash"[tw] OR "debris"[tw] OR "plastic"[tw] OR "odor"[tw] OR "odour"[tw] OR "turbidity"[tw] OR "recreational use"[tw] OR "recreational services"[tw] OR "clean beaches"[tw] OR "beach

	litter"[tw] OR "blue space"[tw] OR "bluescape"[tw] OR "linear park"[tw] OR "visual quality"[tw] OR "loss of habitat"[tw]) OR ("aesthetics"[tw] AND ("perception of aesthetics"[tw] OR "beach aesthetic pollution"[tw] OR "aesthetic quality"[tw] OR "degradation"[tw] OR "value"[tw] OR "Aesthetic Quality Index"[tw])) AND ("human"[tw] OR "human health"[tw] OR "health"[tw] AND ("stress"[tw] OR "mental health"[tw] OR "gastrointestinal obstructions"[tw] OR "gastrointestinal illness"[tw] OR "lacerations"[tw] OR "acute infection"[tw] OR "calm*"[tw] OR "emotional benefits"[tw] OR "cardiovascular"[tw] OR "recreation"[tw] OR "physical activity"[tw] OR "social connections"[tw] OR "social interactions"[tw] OR "obesity"[tw] OR "cancer"[tw] OR "injury"[tw])))
**Web of Science**	((((TS="water" OR TS="waterway" OR TS="marine") AND (TS="clarity" OR TS="color" OR TS="presence of debris" OR TS="litter" OR TS="trash" OR TS="debris" OR TS="plastic" OR TS="odor" OR TS="odour" OR TS="turbidity" OR TS="recreational use" OR TS="recreational services" OR TS="clean beaches" OR TS="beach litter" OR TS="blue space" OR TS="bluescape" OR TS="linear park" OR TS="visual quality" OR TS="loss of habitat")) OR ((TS="aesthetics") AND (TS="perception of aesthetics" OR TS="beach aesthetic pollution" OR TS="aesthetic quality" OR TS="degradation" OR TS="value" OR TS="Aesthetic Quality Index"))) AND ((TS="human" OR TS="human health" OR TS="health") AND (TS="stress" OR TS="mental health" OR TS="gastrointestinal obstructions" OR TS="gastrointestinal illness" OR TS="lacerations" OR TS="acute infection" OR TS="calm*" OR TS="emotional benefits" OR TS="cardiovascular" OR TS="recreation" OR TS="physical activity" OR TS="social connections" OR TS="social interactions" OR TS="obesity" OR TS="cancer" OR TS="injury")))

Supplement Document S3: Individual article narrative summaries

Article 1: *The Association Between Natural Environments and Depressive Symptoms in Adolescents Living in the United States*

Utilizing a cross-sectional approach, Bezold et al. looked at the Growing Up Today (GUTS) cohort from 1999 to examine associations between residential greenspace and bluespace exposure measured by normalized difference vegetative index (NDVI) and self-reported depressive symptoms measured by the McKnight Risk Factor Survey. The authors found directional indication of exposure to greenspace lowering odds of depressive symptoms. The authors also found no indication of exposure to bluespace impacting odds of depressive symptomology.

Overall, Bezold et al. found modest evidence for exposure to green space, but not blue space, lowering the odds of self-reported depressive symptoms among a cohort of U.S. adolescents. Some important limitations to note for this study were the cross-sectional design, unaccountability of quality or access of the green spaces measured, vegetative type not captured by NDVI, and concerns of generalizability beyond the predominantly urban, white cohort of adolescents.

Article 2: *Human health impacts from litter on beaches and associated perceptions: A case study of 'clean' Tasmanian beaches*

Campbell et al. used a cross-sectional approach to evaluate the prevalence of beach litter related injury and beachgoers perceptions of litter. Using the Clean Coast Index and surveying local townships near 9 Tasmania beaches, Campbell et al. reported that just over one-fifth (21.6%) of beach users received a litter related injury. Survey results on perceptions of beach litter revealed that only 12.9% of respondents felt that beach litter posed a major threat to human health, in the form of injuries, instead focusing on the impacts litter has on marine wildlife.

Although the authors attempt to find links between aesthetic qualities of coastal waterways and beaches (i.e., litter) and human health outcomes (i.e., injury) they were limited by the small sample size (n=173) and descriptive statistical analysis. In addition, sampling, self-response, and generalizability concerns all remain. Still, this study provides an important first step to linking elements of aesthetic quality to impacts on human health.

Article 3: *Are our beaches safe? Quantifying the human health impact of anthropogenic beach litter on people in New Zealand*

Expanding on their work in 2016 (see Article 2 above), Campbell et al. looked comprehensively and quantitatively at the impacts of beach litter on injury. Using a cross-sectional design, the authors pulled insurance claims from New Zealand's nationalized insurance system via the Accident Compensation Corporation (ACC) to calculate the number of claims from 2007-2016 filed for injury related to anthropogenic beach litter (ABL). From a total of 161,261 claims the authors utilized linear regressions and analysis of variance (ANOVA) to conclude that ABL related injuries pose a serious threat to beachgoer's health across several demographic covariates (i.e., age, ethnicity, sex, locality, etc.). The authors do note that children 0-14 years of age were at increased risk of ABL related injuries.

While this study was a more robust assessment of the impacts of beach litter on injury than the prior study [18], the authors were unable to relate the amount of beach litter (i.e., the aesthetic degredation) to the risk of injury. The concerns of generalizability are ameliorated in this large and nationally representative study for New Zealand.

Article 4: *Residential green space and birth outcomes in a coastal setting*

Glazer et al. utilized a cross-sectional approach to evaluate residential exposures to green and blue space on the birth outcomes of preterm birth (PTB) and small for gestational age (SGA). Using Rhode Island birth records and NDVI measures the authors used radial buffers around participant addresses to determine exposures to green space, blue space, and recreational facilities. With a sample of 61,460 births, the authors performed logistic regressions for whether a participant was within 500 or 1000 meters of a blue space and/or recreational facility. NDVI was assessed via mean pixel values within a 500-meter buffer and evaluated by IQR increase. After individual and neighborhood level adjustments the authors conclude that exposure to green, blue, or recreational spaces does not reduce risk of preterm birth or risk of low birth weight.

Glazer et al. use multiple measures to examine associations of birth outcomes with exposures to green, blue, and recreational spaces. The authors attempt to draw a link between exposure to "natural" spaces and human health outcomes (PTB and SGA). However, this study does not account for the quality of these spaces, their accessibility beyond proximity, or seasonality. Still, the emphasis placed on socioeconomic adjustment is important since residing near parks and waterways is often correlated to household income and wealth, which can itself improve health outcomes.

Article 5: *Natural environments and suicide mortality in the Netherlands: a cross-sectional, ecological study*

Helbich et al. examined associations between municipality-level green and blue space exposure on risk of suicide using a cross-sectional approach and data from the Dutch Land Use database and the national suicide register from 2005-2014. Exposure was defined by the proportion of area within each municipality designated as green or blue space and was then standardized into low, medium, and high exposure groups. The authors used Bayesian hierarchical Poisson regressions with a sample of 16,105 suicides, and adjustments for gender, divorce, unemployment, housing values, distance to nearest general practitioner, voter alignment, and urbanicty. The authors report a protective effect for exposure to green space, but not blue space, on suicide risk at the municipality-level.

Using national databases and robust modeling methods allowed Helbich et al. to evaluate the role of green and blue space exposure on a relatively rare human health outcome: suicide. Yet, this municipality-level analysis of green and blue spaces did not capture the quality, particularly aesthetic quality, of these spaces. Additionally, there is no data regarding access, safety, or type of greenery or type of water body (i.e,. pond versus river).

Article 6: *Using deep learning to examine street view green and blue spaces and their associations with geriatric depression in Beijing, China*

Using machine learning techniques, Helbich et al. sought to evaluate the impacts of green and blue space exposure on older adult persons and to compare the robustness of machine learning greenspace measures with conventional land-use and NDVI based

approaches. A cross-sectional survey captured 1,190 participants 60 years or older residing in the Haidian district of Beijing China. Participants were assessed using the Geriatric Depression Scale (GDS-15). The authors then captured street view images from Tencent (a Google Maps equivalent for China) in 100-meter segments around the participant's address. Data was also pulled from NDVI and GlobeLand30 (land cover database for China) to compare remote sensing methods to the machine learning street view. A fully convolutional neural network parsed street view images to derive green pixels from non-green pixels, eventually resulting in averages of "greenness" per neighborhood. Chi-square testing, Spearman correlations, and multilevel linear models were utilized to conduct analyses on risk of depressive symptoms, agreement, and fit between exposure modeling approaches. Covariates of age, gender, education, ethnicity, marital status, party membership, Hukou status, functional ability, and physical health status were included in various adjustment schemes. The authors conclude that street view modeling passed robustness tests and match well findings from more typical methods of green space measurement. They further conclude their results are in agreement with other studies looking at green and blue spaces as protective factors against risk of depression among the elderly.

Although unique in its measurements of green and blue space, this article does not capture aspects related to quality, access, safety, and ecology of the spaces. Without these elements, it is difficult to infer the aesthetic quality of the spaces beyond increasing or decreasing values of "greenness". Similar to many of the studies, this study's cross-sectional approach limits its ability to measure either degredation or restoration of spaces to investigate how changes to spaces impact the health of those who reside near the spaces or utilize them regularly.

Article 7: *Neighbourhood blue space, health and wellbeing: The mediating role of different types of physical activity*

Pasanen et al. conducted a study to evaluate the mediating role of physical activity with residential exposure to blue space. Using data from the Health Survey for England (sample of 21,097) the authors developed path models to examine associations between different kinds of self-reported physical activity, exposure to coastal and freshwater sources (measured by linear proximity), and self-reported general and mental health via general health questionnaire (GHQ-12). The design controlled for green space density and a multitude of individual and area-level socioeconomic factors in addition to standard demographic covariates. The authors used maximum likelihood estimation to generate estimates for changes in self-reported health. Pasanen et al. conclude that living nearer the coast is associated with better self-reported general and mental health. Additionally, the authors state that this association was partially mediated by physical activity (primarily walking), but not by other forms of physical activity (i.e., watersports). Lastly, the presence of freshwater in a neighborhood was associated with better mental health; however, this association was not mediated by any physical activity.

Pasanen et al. parsed out the specific impact of exposure to blue space on human health with robust adjustments for socioeconomic measures, greenspace densities, and mediation of physical activity. Still, the study did not account for the aesthetic quality of the green or blue space. Further, the health outcomes were self-reported, which limits insight into specific pathologies. Lastly, the cross-sectional design limits the ability to assess changes to the spaces or the people who reside around them.

Article 8: *Effects of freshwater blue spaces may be beneficial for mental health: A first, ecological study in the North American Great Lakes region*

Pearson et al. sought to evaluate the impact of blue space exposure on mental health, principally hospitalization for anxiety/mood disorder. Blue space exposure was defined as Euclidean distance to and area of overlap averaged by zip code. The outcome of hospitalization for mood/anxiety disorder was pulled from Michigan Inpatient Database (MIDB) with more than 30,000 hospitalizations in the study year (2014). Covariates age, ethnicity, day and month of hospitalization, median income, and population density were included in analysis. The authors then applied a negative binomial regression to estimate associations between blue space exposure (proximity and density) with hospitalization for mood/anxiety disorder. The authors report a small protective effect for distance to a Great Lake with an inverse association for small inland lakes and mood/anxeity disorders. Additionally, percentage of area covered by inland lakes was protective for all lake sizes with mood/anxiety disorders.

This study adds to the evidence for salutogenic effects of blue space exposure but did not account for aesthetic quality. Particularly in the Great Lakes region where large ecological restoration efforts have been undertaken, it is relevant to measure the changes to green and blue spaces, their ecologic quality, perceived quality, and how those relate to discrete human health outcomes.

Article 9: *The effects of naturalness, gender, and age on how urban green space is perceived and used*

Sang et al. utilized a cross-sectional survey design to investigate how perceptions of urban green space naturalness differed across gender and age and how those differences impacted use. The authors gathered 1,347 self-reported responses to the WHO's ten question well-being index from households near six different urban green spaces in Gothenburg, Sweden. The authors used descriptive statistics, ANOVA, and MANCOVA analyses to test various relationships. The authors reported that higher perceived naturalness was positively related to more activities, higher aesthetic value, and higher self-reported wellbeing. Sang et al. found that regardless of perceived naturalness women reported higher aesthetic value in green spaces than men and that older adults reported more green space related activities than young adults. The authors conclude that the benefits of perceived naturalness of green space should discourage city planners from "parkification" and encourage them to leave more natural areas intact near residential zones.

Sang et al. measured an important element of aesthetic degredation: perceived aesthetic value. However, there is still no objective measure of quality, which limits the ability to ecologically define naturalness. Similar to the other studies, the cross-sectional design does not easily lend itself to measuring changes to spaces through remediation efforts or social campaigns to increase access and utility. Additionally, the study method did not permit the authors to determine precise pathologies or health outcomes.

Article 10: *Designing urban green spaces for older adults in Asian cities*

From December 2016 to March 2018, Tan et al. surveyed 326 older adult park goers across 31 different urban green spaces in Hong Kong, China and Tianan, Taiwan. Participants completed the SF-12v2 health survey, which is designed to measure general mental and physical health. Participants also provided information about their perceptions of the green space. The authors used Spearman rank correlations and moderation analyses. The authors found that duration of visit to an urban green space was positively correlated with self-reported mental health and social function. The authors also found that the perceived safety, plant diversity, and maintenance of the park were associated with perceived aesthetic quality. Tan et al. concludes that these results should inform how to plan and design urban green spaces to improve access and aesthetic quality, two elements preferred by older adult park goers.

Tan et al. draws important associations between aesthetic quality, perceived safety, and access with self-reported health measures. This study does attempt to capture elements of aesthetic quality and interpret the impact it has on health among older adult park goers. The limited statistical treatments and generalized health outcomes provide a useful analysis, but the study cannot address the mechanistic role of aesthetic quality or aesthetic degredation.

Article 11: The neighborhood effect of exposure to blue space on elderly individuals' mental health: A case study in Guangzhou, China.

Chen and Yuan utilize a cross-sectional design to assess the relationship between remote-sensed blue space exposure and generalized mental health. The authors used a population size sample technique to narrow in on various neighborhoods of Guangzhou that had sufficient numbers of older adult residents and exposure characteristics of interest (i.e. sufficient coverage of blue space). A total of 966 questionnaires were collected to assess respondent's mental health via the mental health parameters of the SF-36 questionnaire. Residential exposure to bluespace was assessed with multiple measures. The Normalized Difference Water Index (NDWI) was used to capture remotely sensed blue space area coverage in a 1km buffer around the participant residence. Quality of blue space was captured using landscape fragmentation and a patch separation index, which is designed to account for the loss of habitat ecological function that accompanies highly fragmented natural spaces. Additionally, elements of visit characteristics like duration of visits and activity were also collected. The authors also evaluate four possible mediators: air quality, stress, physical activity and social

contact. Multilevel linear models were the primary methods of evaluating associations and included a variety of individual and demographic covariate adjustments. Sensitivity analyses also used propensity score-matching to examine possible selection bias and robustness of associations. The authors report an association between mental health and bluespace exposure, even after accounting for mediators to conclude that neighborhood blue space exposure is protective for older adult's mental health and is partially mediated by pollution, stress, and social contact.

Chen and Yuan found important and robust associations between residential blue space exposure and older adult self-reported mental health. This study went to great lengths to try and accurately capture the exposure beyond a binary present or absent. Quality of the blue space, including ecological function, was collected and several mediation analyses evaluated the role of key potential mediators to draw robust conclusions of the link between blue space exposure and older adult mental health. Still, the cross-sectional design and generalized self-report based mental health outcome are weaknesses of this study where longitudinal designs and more precise outcomes would have further elucidated possible mechanisms. Overall, this study provides strong evidence for a link between blue space exposure and generalized mental health. Further evaluations of aesthetic quality (i.e. ranking of blue spaces, before vs. after remediation efforts, etc.) and more precise outcomes (diagnosis of disease) should be looked at for future studies.

Article 12: Green and Blue Space Availability and Self-Rated Health among Seniors in China: Evidence from a National Survey

Lin and Wu conducted a cross-sectional analysis to investigate associations between neighborhood level green and blue space exposure and self rated health. Green space exposure consisted of 1km buffer Landsat derived Normalized Difference Vegetative Index (NDVI) values and a linear distance to nearest park measure. Blue space was similarly derived, consisting of 1km buffer area coverage and linear distance to nearest blue space. Self-rated Health was collected via the 2011 Chinese Social Survey (CSS) from a one-item measure of overall health. In total 1773 participants 60 years or older were identified and included in analysis. Various individual covariates were also collected from the CSS and additional neighborhood-level covariates (i.e. population density, major roads, etc.) were also included in analysis from other data sources. The main analyses utilized ordinary least squares regression to assess associations. The authors also conducted a series of sensitivity analysis to assess potential modification and robustness of associations. Overall the authors report insignificant or modest findings, concluding a lack of evidence, in this sample, for green and blue space exposure to improve older adult self-reported health.

Lin and Wu's study is an important investigation of the green and blue space association with health, using a national survey. For the purpose of this review, the authors did not include a measure of quality (aesthetic or otherwise) for the green or blue spaces. Further, the highly generalized one-item self-reported health outcome is likely not specific enough to address

mechanistic questions surrounding green and blue spaces. Similar to several studies included in this review, failing to address aesthetic and ecological differences among natural spaces is to assume a homogeneity that all natural spaces are essentially the same. Similarly, using highly generalized health endpoints fails to establish a mechanistic pathway by which natural spaces might be impacting health.

Article 13: The effect of urban nature exposure on mental health — a case study of Guangzhou

Liu et al. conducted a cross-sectional study to assess the relationship between self-reported mental health with green and blue space exposure. Using mixed-mode surveys the authors collected data for 933 participants. Mental health was assessed via the Mental Health Inventory (MHI-5) to capture depressive symptoms. Green space exposure was assessed by network distance of the nearest park with blue space captured by area of coverage within a series of buffers (300m-1000m) as well as Euclidian distance from residence to nearest body of water. Various demographic and socioeconomic covariates were included in analysis. The authors utilized regression to assess associations with several additional sensitivity analysis to explore potential modification and robustness. The authors report significant associations between reported mental health and nearest park and blue space, adding to the body of evidence suggesting salutogenic benefit to green and blue space exposure.

This work by Liu et al. does provide further evidence for a possible salutogenic benefit to residential green and blue space exposure. The measures of residential green and blue space are valid for the question the authors set out to answer. However, to better understand possible mechanisms it is imperative that green and blue space studies begin to attempt to capture elements of ecology and aesthetics. It is also imperative that these studies begin to look at more specific outcomes, such as an actual diagnosis of depression, in place of self-reported symptomology.

Article 14: General health and residential proximity to the coast in Belgium: Results from a cross-sectional health survey

Using data from the Belgian Health Interview Survey, Hooyberg et al. examined associations between residential proximity to coastlines and general health. A total of 60,939 survey response were included in this analysis with general health assessed via a single question: "How is your health state in general?" Distance to coastline was calculated by network distance and defined in eight categories ranging from 0-5km to more than 250km. The authors utilized linear regression models with various individual and demographic covariate adjustments to determine associations. The authors also conducted several sensitivity and mediation analysis of hypothesized mechanisms (physical activity, social interaction, and air pollution). The authors report a protective association for self-report general health and coastal proximity for those living closes to a coastline. Results from mediation analyses did not reveal that the hypothesized mechanisms accounted for the observed relationship between general health residential proximity to the coast. Hooyberg et al. conclude that there is modest evidence

for a link between living near a coast and general health, although mechanisms of action are still unclear.

Hooyberg et al. builds upon existing literature examining the coastal proximity-health relationship and adds important mediation analyses to look at specific hypothesized mechanisms. Similar to many studies in this review, the generalized self-report outcome and cross-sectional design, while sufficient for the research question set out for the authors, should be bolstered for analysis of degredation. Assuming that the entire Belgian coastline is not identical, accounting for variation in aesthetic quality, access, safety, etc. would also be preferable in future analyses. Longitudinal designs would aid in answering questions around mechanisms. For example, how much coastline exposure is beneficial as a response to frequency of visits or length or visits. Further, there is an underlying assumption that coastline exposure is always a benefit. This ignores issues related to aesthetics, which may in fact be harmful (i.e. oil spills, algae blooms, storm-events, etc.). Overall, Hooyberg et al. adds to the evidence for possible benefits to residential proximity to a coastline, but future work should investigate the role of aesthetics and degraded environments on specific outcomes to aid in parsing out possible mechanisms.

Article 15: Different types of urban natural environments influence various dimensions of self-reported health

Jarvis et al. conducted a cross-sectional study to evaluate the relationship between residential exposure to natural spaces and self-reported health in Vancouver, Canada. The authors utilized responses from the Canadian Community Health Surveys (CCHS) to assess generalized health outcomes like self-reported general health, mental health, and common mental disorder from survey response. Environmental data was assessed at the postal code level using combinations of remote-sensed data (Google Earth, NDVI, LiDAR) to quantify land cover types over a variety of buffer distances (250m, 500m, and 1000m). Several individual and demographic covariates were included in analysis to adjust for potential confounders. The authors utilized logistic regression models to determine associations. Additional sensitivity analyses examined the robustness of models and comparisons of land cover based assessments against NDVI measures. Although this analysis included nearly 2 million responses, the authors report not finding significant associations between greenspace exposure and self-reported health. Jarvis et al. go on to conclude that the land cover type influences the association between health and natural exposures. For example, water and some vegetation was associated with reduced odds of poor self-reported health, while paved surfaces increased the odds.

Overall, this study adds to a growing body of evidence that land cover types do influence health. Jarvis et al. explore the influence of several land cover types and not just blue or green space exposure, nearing the concept that not all spaces start as neutral (i.e. paved surfaces were shown to be harmful). This is a step toward assessing aesthetics and degredation. Still, the self-report outcomes and cross-sectional design make hypothesis generation of possible mechanisms more difficult. Still, this large sample study does provide compelling evidence for

the role land cover plays in human-environment interaction and nears a population-level assessment of aesthetics and degredation.

Article 16: Cross-sectional association between the neighborhood built environment and physical activity in a rural setting: the Bogalusa Heart Study

Gustat et al. utilized data from the Bogalusa Heart Study, unique assessments of built environment and physical activity questionnaires to assess the relationship between physical activity and the built environment in a neglected setting: rural America. Using 1245 participants of the Bogalusa Heart Study, the authors collected information on the rural activity via the Rural Active Living Assessment. This assessmnt uses Google Street view segments to determine the features of any given segment that improve or harm physical activity (i.e. a street that has continuous sidewalk segments promotes physical activity). Physical activity was assessed with the International Physical Activity Questionnaire (IPAQ) to determine the weekly metabolic equivalent minutes for total, leisure time, and transport physical activity. The authors then assessed street segments to assign a built environment (BE) score by looking at features which included pedestrian safety, aesthetics, physical security, land use, destinations, and more. Neighborhood BE scores were then calculated by averages of segment scores, weighted by inverse distance (i.e. segments closer to the residence have greater weight). The authors used generalized estimating equations linear regression, adjusted for several individual and demographic covariates to assess associations between BE score and physical activity. The authors did not find a significant association between overall BE score and physical activity, but did observe associations for some sub-scores and activity. Most notably for this review, leisure physical activity was positively associated with aesthetic score. Gustat et al. also note that the built environment overall was not conducive for physical activity, a problem noted generally in rural America.

This study by Gustat et al. provides critical evidence of the built environment's role in promoting physical activity and does so in rural Louisiana. The unique street view segment based assessments of the built environment leverage modern technologies to more accurately assess the ground-level features that impact human-environment interaction. While it is not entirely clear how the aesthetic score was derived, the positive association between increasing aesthetic score and leisure physical activity is an important finding. The cross-sectional design does not lend itself to study changes to the environment, which would be important to capture degredation or remediation. The outcome of physical activity is an important one, but is more often thought of as a mediator rather than an endpoint in itself. Still, this study gets very close to going beyond binary measures of the environment (i.e. greenspace present or not) to measuring features of the environment at ground level which impact how people perceive and utilized natural spaces.

Article 17: Perceived biodiversity, sound, naturalness and safety enhance the restorative quality and wellbeing benefits of green and blue space in a neotropical city

Fisher et al. conducted a cross-sectional study in Georgetown, Guyana to assess associations between perceptions of green and blue space and self-reported wellbeing. The authors identified several parks and waterways by assessing land cover and other environmental variables in which to conduct surveys. The authors also assessed bird song diversity and bird counts as a measure of ecological function and biodiversity. Participants were asked to complete the questionnaire while in the space with wellbeing assessed by the Positive and Negative Affect Schedule (PANAS). Mixed-effects models were utilized to assesses relationships between perceptions (i.e. sound enjoyment, species richness, etc.) and momentary wellbeing adjusting for a variety of individual and demographic covariates. The authors also conducted several sensitivity and mediation analyses to examine robustness of association, explore effect modification, and possible mediators. Overall, the authors conclude that restorativeness of green and blue spaces is greater if the individuals perceives the site as safe, species rich, and natural.

This study by Fisher et al. does suffer from many of the pitfalls noted in other articles of this review such as a cross-sectional design and generalized health outcome. However, this study is the more robust assessment of the environment included in this review. The authors went to great length to determine the safety, naturalness, and health of each green and blue space not only in people's perception of this space, but by using ecological measures (i.e. bird song diversity as a proxy of biodiversity). The implication being, the more biodiverse a space the healthier the space's ecology. The authors did find that spaces perceived as more natural were preferred by space users and associated with better self-reported wellbeing. Fisher et al. provide early evidence that aesthetic quality is linked to human health and future studies could examine changes to these spaces to capture elements of degredation or remediation.

Article 18: Greenspace Inversely Associated with the Risk of Alzheimer's Disease in the Mid-Atlantic United States

This study by Wu and Jackson used Center for Medicaid and Medicare data (CMS) and remotely sensed land cover data to examine associations between zip code area greenspace and Alzheimer's Disease (AD). CMS data from 1999 to 2013 identified individuals 65+ who had ICD classification of AD. Land cover data was derived from aerial photography and defined land into six major categories: water, trees, herbaceous, barren, impervious, and roads. $PM_{2.5}$ data was also included from EPA monitor data. All data was aggregated up to the zip code level. The authors utilized zero-inflated Poisson models to examine associations and included several area-level covariates (i.e. median household income, population density, road density, etc.) in models to adjust for potential confounding. Seasonality, sex, and race were also assessed with stratified analyses. Wu and Jackson reported positive associations for exposure to $PM_{2.5}$ and AD risk and negative associations for exposure to greenspace and water with AD risk. These results were consistent across season, sex, and race. The authors conclude that there is further evidence to suggest harm from $PM_{2.5}$ exposure and AD risk and protective benefit for green and blue space exposure.

This study by Wu and Jackson does add to the respective bodies of evidence for the harm of $PM_{2.5}$ exposure and the benefits of green and blue space exposure in a large ecological analysis. In the context of this review the key strength of this study was the specific health endpoint, diagnosis of Alzheimer's Disease. The zip code aggregated measures of green and blue space, while adequate for this ecological analysis, cannot accurately assess the discrete aesthetics and ecological difference between the various green and blue space in any given zip code.

Article 19: The Restorative Health Benefits of a Tactical Urban Intervention: An Urban Waterfront Study

Roe et al. leveraged a crossover design to examine pre and post walk measures of mood and heart rhythms with exposure to a section of coastal walkway in West Palm Beach Florida. 23 participants were randomized into two groups and then asked to walk along a section of walkway under differing conditions. The experimental condition added various aesthetic features, such as shading, seating, and window frames designed to act as "fasciation frames". The control condition was the walkway as it normally exists. The authors used the Mood Adjective Checklist and other perception-based surveys to assess participant moods/perceptions before and after the two conditions. The authors also used real-time activity data from wearable monitors to determine heart rhythms as a measure of stress. Overall, the results showed meaningful differences for the intervention vs. control conditions for the psychological measures. The authors conclude that these differences point to increased psychological restoration from the aesthetic additions of the intervention condition. Roe et al. further conclude that they could not detect a difference in heart rhythms between the two conditions, in part due due to the control condition not being neutral.

Roe et al.'s work is a real-world example of changes to aesthetic condition having measurable changes in human health outcomes. The small aesthetic additions to the walkway have measurable changes on reported psychological endpoints and perceptions about the space. While the heart rhythm findings were inconclusive, the utilization of highly temporally resolved wearable monitors was another great addition. By using real-time data collection, the short-term impacts of this minor aesthetic change were measurable. Roe et al. also demonstrates a key issue with aesthetics research, by showing how the control condition is not neutral. For various reasons (heat, noise, safety concerns, etc.) participants indicated being more stressed under the control condition, which is one possible explanation for the lack of findings for heart rhythm data. Future aesthetics research can build from the work of Roe et al. by increasing sample size, altering the conditions of the experiments, and looking at conditions as non-neutral going forward.
